# The Mannose Receptor Mediates Dengue Virus Infection of Macrophages

**DOI:** 10.1371/journal.ppat.0040017

**Published:** 2008-02-08

**Authors:** Joanna L Miller, Barend J. M deWet, Luisa Martinez-Pomares, Catherine M Radcliffe, Raymond A Dwek, Pauline M Rudd, Siamon Gordon

**Affiliations:** 1 Sir William Dunn School of Pathology, University of Oxford, Oxford, United Kingdom; 2 School of Molecular Medical Sciences, Institute of Infection, Immunity and Inflammation, University of Nottingham, Queen's Medical Centre, Nottingham, United Kingdom; 3 Glycobiology Institute, Department of Biochemistry, University of Oxford, Oxford, United Kingdom; University of Florida, United States of America

## Abstract

Macrophages (MØ) and mononuclear phagocytes are major targets of infection by dengue virus (DV), a mosquito-borne flavivirus that can cause haemorrhagic fever in humans. To our knowledge, we show for the first time that the MØ mannose receptor (MR) binds to all four serotypes of DV and specifically to the envelope glycoprotein. Glycan analysis, ELISA, and blot overlay assays demonstrate that MR binds via its carbohydrate recognition domains to mosquito and human cell–produced DV antigen. This binding is abrogated by deglycosylation of the DV envelope glycoprotein. Surface expression of recombinant MR on NIH3T3 cells confers DV binding. Furthermore, DV infection of primary human MØ can be blocked by anti-MR antibodies. MR is a prototypic marker of alternatively activated MØ, and pre-treatment of human monocytes or MØ with type 2 cytokines (IL-4 or IL-13) enhances their susceptibility to productive DV infection. Our findings indicate a new functional role for the MR in DV infection.

## Author Summary

Dengue disease and its severe manifestations are a growing public health concern, with a third to half the world's population living in dengue-endemic areas. In recent years there have been significant advances in understanding dengue virus (DV) interactions with target cells such as macrophages, dendritic cells, hepatocytes, and endothelial cells. Interaction with and infection of these cells leads to the production of new virions as well as immune mediators, which can shape the course of the subsequent immune response. The vascular leakage associated with dengue haemorrhagic fever is believed to be immune mediated. Our work on the interaction of DV with human macrophages has led to two major findings; first, we have identified that the macrophage mannose receptor is important for mediating the infection of human macrophages by DV, and second, that the type 2 cytokines IL-4 and IL-13 enhance macrophage susceptibility to DV infection. DV–receptor interactions are of critical importance for understanding not only the mechanisms of entry, but also the biology of infection and the pathogenesis. Understanding the immunopathogenesis of dengue disease is crucial to the development of both a safe dengue vaccine and therapeutic inhibitors of early DV replication.

## Introduction

Dengue is the most prevalent mosquito-borne viral disease worldwide and in the past 40 years has undergone a global resurgence such that almost half the world's population are currently living at risk in dengue-endemic areas [1]. There is a spectrum of disease severity following dengue virus (DV) infection that in its more severe forms results in dengue haemorrhagic fever (DHF) and shock syndrome. The resultant morbidity and mortality, and subsequent considerable economic burden, make the development of a safe and effective vaccine imperative. DV pathogenesis is complex and multifactorial [2], and macrophages (MØ) are thought to play an important role in disease both as primary targets of viral infection and as a source of immunomodulatory cytokines. The four serotypes of DV (DV1-DV4) bind to a number of opsonic and non-opsonic receptors on cells of the mononuclear phagocyte lineage including DC-SIGN [3,4], glycosaminoglycans [5], and when in complex with specific antibody, Fc and complement receptors [6].

MR is a multi-domain protein that is composed of a cysteine-rich (CR) domain which has lectin activity and binds to sulphated sugars, a fibronectin type-II (FNII) domain that mediates binding to collagen [7] and eight C-type-lectin-like domains (or carbohydrate-recognition domains, CRD). The fourth CRD mediates most of the specificity of these domains for glycans terminating in mannose, fucose and N-acetyl glucosamine. In addition to many endogenous ligands, MR binds to bacteria (e.g. Mycobacterium tuberculosis), fungi (e.g. Pneumocystis carinii) and viruses (e.g. HIV). MR is constitutively internalized from the plasma membrane by clathrin-mediated endocytosis and recycled back to the cell surface. Intracellular targeting is mediated by a tyrosine-based motif in the cytoplasmic tail, although it contains no recognised signalling motifs (for a comprehensive review see [8]). DC-SIGN, a lectin with similar sugar specificity to that of the MR, can mediate DV attachment to dendritic cells [3,4]. Even though DV binding to DC-SIGN on these cells is important for attachment, DC-SIGN-mediated viral endocytosis is not required for DV entry [9].

While the immune response to viruses is classically described as Th1 mediated, the literature in the case of DV suggests that this may not be absolute. IgE (characteristic of a Th2 environment) has recently been shown to be elevated in the acute stages of DV infection [10] and at defervesence [11]. A microarray study of whole blood gene expression during secondary DV infection has shown early upregulation of IL-13 transcripts in acute samples from DHF patients [12]. This suggests that a Th2 response may be occurring in patients at some stages of infection. Indeed, studies of IL-12, IL-13 and TGFβ cytokine levels in DHF patients suggest that the DV response shifts from a Th1-dominant response to a Th2-biased response during disease progression [13,14]. The immune responses in infants/neonates differ qualitatively from those of adults, with the immature immune system having a bias towards Th2 rather than Th1 immune responses, presenting a particularly relevant challenge for pediatric DV vaccination [15].

MØ are profoundly influenced by the cytokine profile in their immediate environment. Functionally diverse subsets of alternatively or classically activated mononuclear phagocytes can develop in an immune response. Exposure of MØ to IL-4 or IL-13 elicits an ‘alternate type of activation’, as opposed to the classical activation induced by IFNγ [16]. These alternatively activated cells have been implicated to have regulatory functions in cellular and humoral immunity by affecting the balance of pro- and anti-inflammatory reactions [17]. Protein expression studies and transcriptional profiling have shown that IL-4 induces upregulation of several receptors, including the mannose receptor (MR) on monocytes and MØ [18,19].

In this study we show that MR binds to DV grown in mosquito cells and to recombinant mammalian cell–produced DV envelope glycoprotein. A recombinant MR fusion protein (CRD4–7-Fc) was shown to recognize DV envelope (E) protein in ELISA and blot overlays, and binding was inhibited by mannose, fucose and EDTA. The presence of MR on transfected cells is sufficient to confer DV binding. DV infection of MØ was blocked by antibodies against the human MR suggesting that it is a novel functional receptor contributing to DV infection. We also show that pre-treatment of primary human monocytes with Th2 cytokines (IL-4/IL-13), which upregulate MR expression, increases their susceptibility to DV infection in vitro. Better understanding of receptor/s and entry pathways mediating infection in humans could be crucial to the design and safety of a dengue vaccine.

## Results

### Soluble MR Binds Mosquito Cell–Derived DV and Recombinant Soluble E Protein

**Figure 1:**
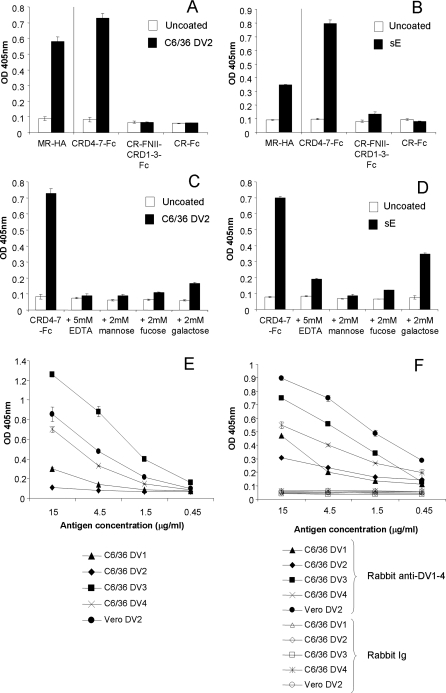
MR Binding to DV Is Mediated by the CRD4–7 (A) Binding of MR extracellular domain (MR-HA) and chimeric MR fusion protein constructs to mosquito cell–derived (C6/36) NGC strain DV2 and (B) soluble E protein (sE) produced in a human cell line, detected by ELISA. Closed bars, DV antigen; open bars, uncoated wells. The MR-HA protein was detected with an anti-murine MR antibody (MR5D3). Binding of the Fc fusion proteins to the DV/sE was detected with an anti-Fc antibody. (C) Inhibition of CRD4–7-Fc binding to mosquito cell–derived semi-purified DV2 and (D) sE, detected by ELISA as above. (E) Binding of CRD4–7-Fc to all four serotypes of DV, detected with an anti-Fc antibody. Strains examined were mosquito cell (C6/36)–derived virus of DV1 (Hawaii), DV2 (NGC), DV3 (H-87) and DV4 (H-241), and Vero cell–grown DV2 (16681). Differential binding to the serotypes may reflect coating levels, as indicated by (F) binding of rabbit anti-DV1–4 antibody. Normal rabbit immunoglobulin (RIg) was included as a control and both were detected with an anti-rabbit antibody. The low levels of CRD4–7-Fc binding to C6/36-grown DV2 in this panel reflect a shorter development time than in the other panels. Data are expressed as mean and SD of triplicate wells. Representative data from two to five independent experiments are shown. Recombinant fusion proteins contain the cysteine-rich (CR) domain, fibronectin type-II (FNII) domain, and various carbohydrate-recognition domains (CRD) of the MR.

The ability of MR to bind DV antigen produced in mosquito (C6/36) and human (293T) cells was examined. ELISA wells were coated with semi-purified C6/36-grown DV2 or recombinant soluble E (sE) protein produced in the endothelial kidney cell line 293T (see below for characterisation of this reagent) and probed with the entire extracellular region of the murine MR expressed with an HA tag or recombinant truncated forms of the murine MR with human Fc tags. MR-HA bound to purified mosquito cell–derived DV2 (Figure 1A) and to sE (Figure 1B), and binding was mediated specifically by CRD4–7 and not the CR or the FNII domains. Binding of CRD4–7-Fc to both C6/36-grown DV2 (Figure 1C) and sE (Figure 1D) was inhibited by 2mM d-mannose, 2mM l-fucose, and to a lesser extent 2mM d-galactose, and depended on the presence of divalent cations. This is consistent with the known sugar specificity and calcium dependence of the MR CRD4–7 domains.

We extended the study by investigating the binding of CRD4–7-Fc to mosquito cell–derived virus of the other 3 DV serotypes (DV1, DV3 and DV4) and to mammalian (Vero) cell–grown DV2 by ELISA. CRD4–7-Fc bound to all four serotypes of DV in a dose-dependent manner (Figure 1E). Binding of the CRD4–7-Fc correlated with the different coating levels of these antigens as determined with an antibody against all 4 DV serotypes (Figure 1F). In addition, we tested whether CRD4–7-Fc and sMR-HA interacted with other flaviviruses. ELISA data showed that both CRD4–7-Fc and MR-HA both bound to Japanese encephalitis virus (inactivated vaccine antigen) and tick-borne encephalitis virus (inactivated, mouse brain-grown) (data not shown).

**Figure 2:**
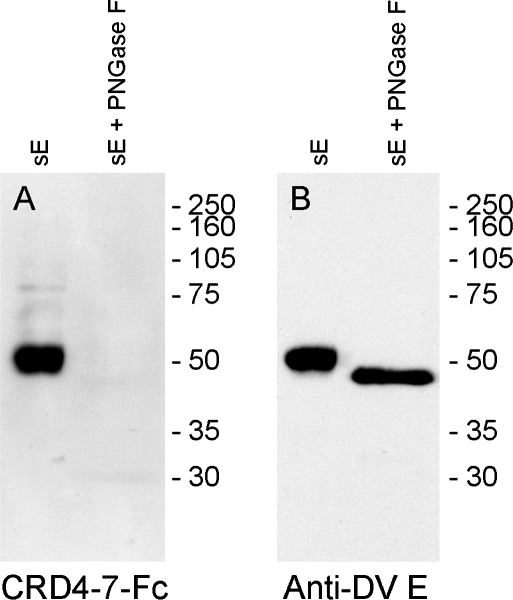
MR CRD4-7-Fc Binds Specifically to sE in Blot Overlay, and Binding Depends on N-Linked Sugars sE and PNGaseF-treated sE were resolved by SDS-PAGE using 10% gels, transferred to nitrocellulose membranes, and (A) probed with MR CRD4–7-Fc. (B) Blots were subsequently stripped and reprobed with the anti-E protein antibody, 3H5.

The specificity of MR CRD4–7-Fc binding to DV sE was further examined by blot overlay. CRD4–7 bound exclusively to a single band that migrated at 52 kDa (Figure 2A, left-hand lane). This band was recognized by the anti-E protein antibody, 3H5, when blots were stripped and reprobed (Figure 2B, left-hand lane), confirming that CRD4–7 binds to DV E-protein. CRD4–7 did not bind sE deglycosylated with peptide:N-glycosidase F (PNGaseF) (Figure 2A, right-hand lane), in contrast to 3H5 that bound to both the native and the deglycosylated forms of the protein (Figure 2B), indicating that CRD4–7 binds specifically to N-linked glycans on sE. Binding of CRD4–7 to sE was inhibited by the presence of either 2mM d-mannose, 2mM l-fucose or 20mM EDTA. The presence of sE on these blots was confirmed by washing blots and reprobing in the absence of inhibitors (data not shown). In addition, CRD4–7-Fc did not bind to unglycosylated domain III of DV E protein produced in bacteria in ELISA experiments (data not shown).

**Figure 3:**
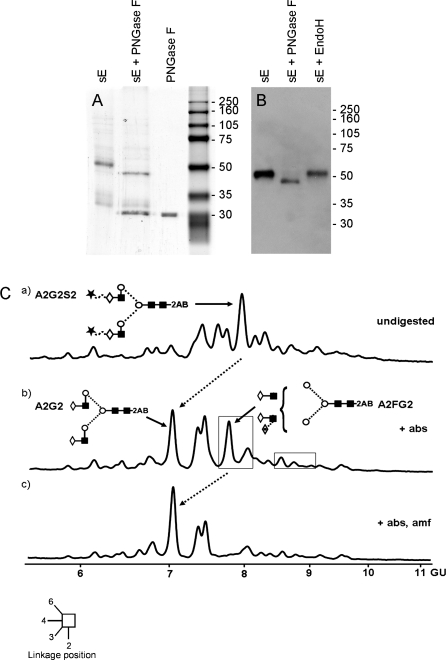
Production and Characterisation of Recombinant Soluble Dengue Virus E-Glycoprotein (A) SDS-PAGE of sE protein preparation resolved on 10% gel and stained with Coomassie Brilliant Blue. Lane 1, sE preparation as eluted from NiNTA-agarose. Lane 2, PNGaseF-treated sE. Lane 3, PNGaseF alone. (B) Western blot of sE before (Lane 1) and after treatment with PNGaseF (Lane 2) or EndoH (Lane 3), resolved under reducing conditions by 10% SDS-PAGE, and probed with the anti-DV E monoclonal antibody, 3H5. (C) NP HPLC chromatograms of the complete pool of 2AB labelled glycans of sE glycoprotein, together with sequential digestions. a) Complete glycan pool, undigested, with structural representation of A2G2S2. b) Glycans digested with Arthrobacter ureafaciens sialidase (abs), which releases α2–6 and 3 linked sialic acids. Boxed sections show peaks containing α1–3 or 4 linked fucose residues, with structural representations of A2G2 and A2FG2. c) Glycans digested with abs and almond meal fucosidase (amf), which releases α1–3 and 4 linked fucose residues. Key: A2, biantennary; G, galactose; F, fucose; S, sialic acid. Filled square, *N*-acetyl glucosamine; open circle, mannose; open diamond, galactose; filled star, sialic acid; open diamond with dot, fucose. The solid lines are β-linkage, dotted lines are α-linkage, and curved lines are unknown linkage.

Given the interaction of DV with MR described above, it was important to characterise the glycans on the human cell–produced sE, especially since we are unaware of any similar analysis in the literature. This reagent is valuable, as its glycan modifications may more closely resemble the patterns found on viral particles produced during infection in the human host compared with baculovirus and E. coli produced molecules. DV E protein has two conserved N-linked glycosylation sites at Asn-67 and Asn-153. Deglycosylation of sE by PNGaseF led to a shift in apparent mobility on SDS-PAGE from 52 kDa to 46 kDa (the predicted molecular weight of sE is 45 kDa), indicating that the protein carries N-linked glycan modifications (Figure 3A and 3B). Conversely, digestion of sE by endoglycosidase H, which cleaves high mannose oligosaccharides, did not result in a mobility shift on SDS-PAGE (Figure 3B). RNAse B was deglycosylated by both enzymes under corresponding reaction conditions as a positive control (data not shown). A more specific glycan analysis by sequential digestion with sialidase, fucosidase and mannosidases (Figure 3C) showed approximately 40% of the glycoforms were sialylated and 25% contained α1–3,4 linked outer arm fucose. There was no evidence of terminal mannose. The glycans were also processed by weak anion exchange (WAX) HPLC before and after sialidase digestion. There were charged glycoforms remaining after sialidase digestion which may be sulphated (data not shown).

### Cell Surface MR Expression Confers DV Binding

**Figure 4:**
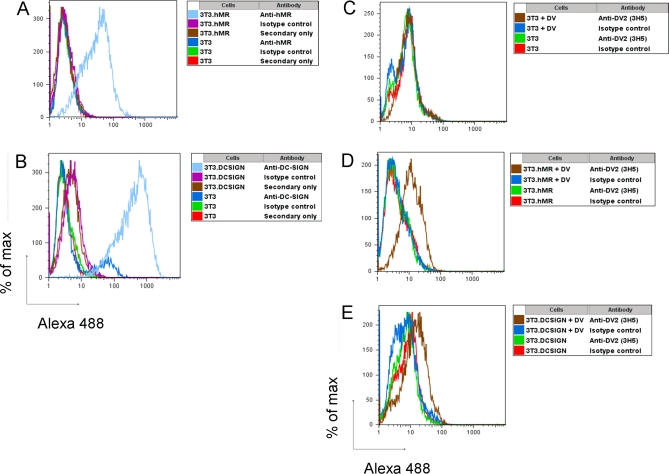
DV Binds to Cells Transfected with Human MR or DC-SIGN The expression levels of (A) MR and (B) DC-SIGN on cells transfected with MR (3T3.hMR), DC-SIGN (3T3.DC-SIGN), or vector only (3T3) were assessed by flow cytometery. Receptor expression was detected with 15–2 (anti-hMR) or 120507 (anti-DC-SIGN) antibodies (pale and dark blue lines). Matched isotype control (purple and green lines) and secondary antibody only (brown and red lines) staining is included. Binding of NGC DV2 to cells transfected with (C) vector only (3T3), (D) MR (3T3.hMR), and (E) DC-SIGN (3T3.DC-SIGN) for 90 min on ice was detected by flow cytometry. The histograms show the binding of anti-DV2 antibody (3H5; brown and green lines) and isotype matched control antibody (blue and red lines). The relative fluorescence intensity was measured by FACSCalibur analysis and the data are normalised and presented as percent of maximum. Representative data from one of two independent experiments are shown.

To further evaluate MR as a potential DV receptor, we examined binding of DV to human MR-transfected 3T3 cells (3T3.hMR). As DC-SIGN has previously been shown to be an important attachment receptor for DV, DV binding to 3T3.hMR cells was compared with binding to 3T3 cells transfected with DC-SIGN. Initially we confirmed expression of the respective receptors on the 3T3 cell surface (Figure 4A and 4B). To assess virus binding, cells were incubated with mosquito cell–grown DV, unbound virus was washed away and DV bound to the cells was detected with the anti-E protein antibody, 3H5 (Figure 4C–4E). Histograms show a clear shift in fluorescent intensity indicating DV binding to cells transfected with either human MR or DC-SIGN. Similar data were observed using anti-pre-membrane glycoprotein (prM) antibody (2H2; data not shown) to detect bound DV. Thus, surface expression of human MR on transfected 3T3 cells was sufficient to confer DV binding.

### Type 2 Cytokines Enhance MØ Susceptibility to DV Infection

A human primary cell culture assay system was established in which we examined the functional role of MR and the effects of cytokines on the susceptibility of mononuclear phagocytes to DV infection. Monocytes were purified from human PBMC fractions and cultured for 2 or 7 d to prepare monocyte-derived MØ (MDMØ) or differentiated into monocyte-derived dendritic cells (MDDC) prior to infection. The percentage of cells infected was quantified by microscopy by staining nuclei with DAPI and viral antigen with the anti-DV2 E protein monoclonal antibody, 3H5. MDDC were more susceptible to infection by DV (percent infected DC: 12.3% +/− 7.1) compared with either 2 or 7 d differentiated MDMØ in the absence of added cytokine (percent infected 2 d MDMØ: 1.8% +/− 0.7; 7 day MDMØ: 1.1% +/− 0.3) from the same donor (3 donors), using a multiplicity of infection of 0.4. The presence of DV non-structural protein in infected 2 d MDMØ using anti-NS1 monoclonal antibodies (obtained from Eva Harris) suggested that active viral replication and de novo viral protein production, rather than mere uptake of viral antigen, was occurring (data not shown). Plaque assays on supernatants from infected primary 2 or 7 d MDMØ cell cultures confirmed the occurrence of productive infection, with viral titres increasing over time and reaching 10^4^ pfu/ml in cell supernatants 2 d after infection of 6 × 10^6^ cells.

**Figure 5:**
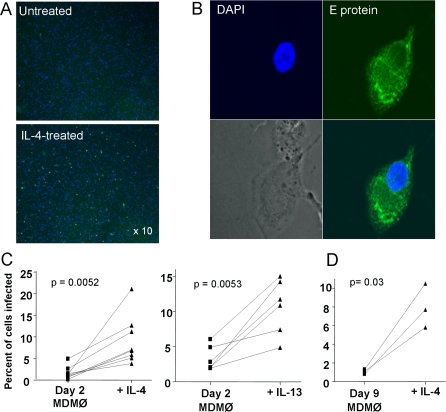
IL-4 and IL-13 Enhance Monocyte and MØ Susceptibility to DV Infection Monocytes isolated from human peripheral blood were either treated with IL-4 or IL-13 or were left untreated for 2 d (day 2 MDMØ) prior to infection with dengue virus. Two days following infection, permeabilized cells were stained with antibody to dengue E protein (green) and nuclei stained with DAPI (blue). (A) Low power image. (B) Single DV-infected cell confocal image showing DV protein distributed throughout the cytoplasm. Image represents a single *x*-*y* section through the middle of a cell. Fluorescence images are shown next to the corresponding transmission image. (C) The percentage of cells infected was counted by microscopy. Each line represents a single donor. (D) Monocytes were matured into MDMØ by 7 d incubation prior to treatment with IL-4 for 2 d (day 9 MDMØ). Cells were infected with DV and stained, and percent infected cells calculated.

Considering that MDDC, which were grown in an IL-4/GM-CSF cytokine cocktail, were infected to a higher degree than the MDMØ, various cytokines were tested for their ability to alter the susceptibility of MDMØ to DV infection by pre-incubation with monocytes for 48 h prior to DV infection (see Table S1 for full list and concentrations). Only the type 2 cytokines IL-4 and IL-13, which utilize a common receptor chain, had a substantial effect on the susceptibility of MDMØ to DV infection in culture (Figure 5A and 5B), increasing the percentage of infected cells from around 1% to between 4% and 21% (Figure 5C). An almost 6-fold average increase in the percentage of cells infected was observed for 8 independent donors (p = 0.005). IL-4 may also contribute to the enhanced degree of DV infection of MDDC, as GM-CSF alone did not increase the level of infection of MDMØ (Table S1).

Neither the age of the cells nor the length of treatment altered the enhancing effects of IL-4. Enhanced susceptibility to DV infection was seen when monocytes were allowed to differentiate into MØ over 7 d, and then treated with IL-4 for 48 h prior to DV infection (day 9 MDMØ, Figure 5D). An 8-fold increase in the percentage of infected cells was observed for 3 independent donors (p = 0.03). Alternatively, monocytes treated for 2–7 d with IL-4 prior to infection with DV all showed similar heightened susceptibility (data not shown). This suggested that the increased number of infected cells was not due to maturation of the cells. A dose response experiment indicated that the enhancement of DV infection of MDMØ could be achieved with as little as 1.5ng/ml IL-4 (data not shown).

**Figure 6:**
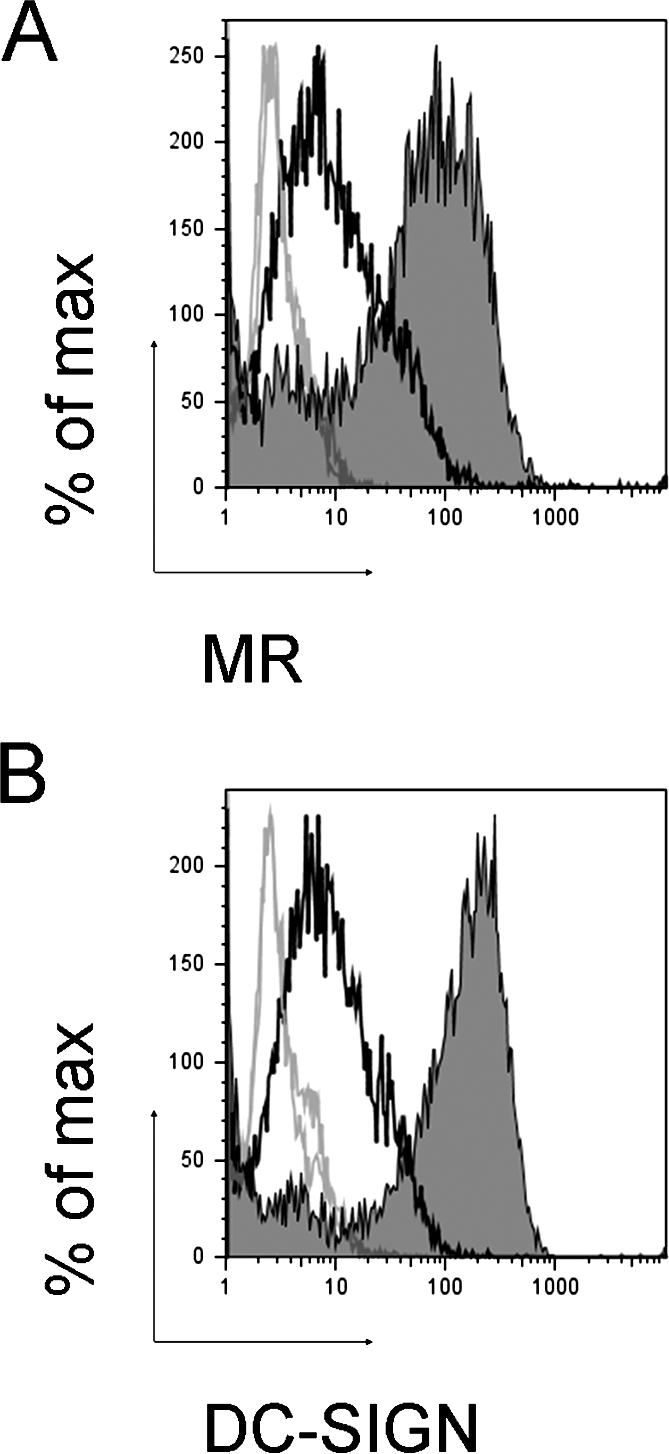
IL-4 Treatment Enhances Surface Expression of MR and DC-SIGN on Human MDMØ The effects of 2 d IL-4 treatment (25ng/ml) of human monocytes on surface expression levels of (A) MR and (B) DC-SIGN were assessed by flow cytometery. Receptor expression was detected with 15–2 (anti-hMR) or 120507 (anti-DC-SIGN) antibodies (black lines) on untreated cells (open histogram) and IL-4-treated cells (filled histogram). Matched isotype control (grey lines) staining is included. The relative fluorescence intensity was measured by FACSCalibur analysis and the data are normalised and presented as percent of maximum. Representative data from one of eight donors are shown.

The interaction of DV with primary cells appears to be multifactorial, as we found variability between donors and receptor expression level. Surface expression of both MR and DC-SIGN is upregulated on MDMØ by IL-4 treatment (Figure 6A and 6B), consistent with previous data for DC-SIGN on human monocytes [20] and for MR on primary mouse MØ [19,21]. While the fold increase in surface MR levels following IL-4 treatment (4.1–fold +/−1.3) parallels the fold increase in the percent of infected cells (4.6-fold +/− 1.4), from analysis of a limited number of donors no clear correlation can be drawn between the two. This is also true of the relationship between upregulation of surface DC-SIGN expression (7.7-fold +/− 0.9) on the same IL-4-treated cells and increase in the percent of infected cells (mean of 4 donors; data not shown).

### Anti-MR Antibodies Block DV Infection in Human MØ

**Figure 7:**
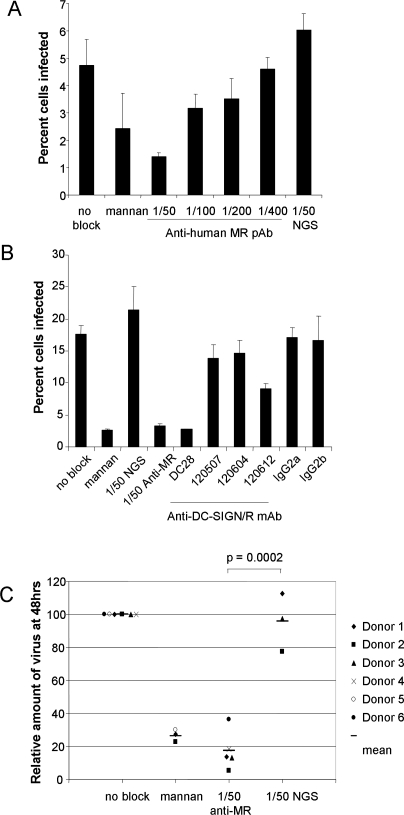
Anti-MR and Anti-DC-SIGN Antibodies Inhibit the Ability of DV2 to Infect IL-4-Treated Monocytes (A) IL-4-treated human monocytes were incubated in triplicate wells with medium alone (no block), 2mg/ml mannan, titrations of goat anti-human MR antiserum, or with normal goat serum (NGS). Treated cells were infected with mosquito cell–grown 16681 DV2 at a multiplicity of infection of 0.5 in the presence of these inhibitors, incubated for 48 h and fixed. (B) Monocytes were treated in triplicate wells, as above, including blocking with monoclonal antibodies specific for DC-SIGN (120507), DC-SIGNR (120604), and both DC-SIGN and DC-SIGNR (DC28 and 120612) or isotype controls (all at 5ug/ml) prior to infection with mosquito cell–grown 16681 DV2 at a multiplicity of infection of 0.04 in the presence of these inhibitors. Following fixation, cells were immunolabelled with anti-DV E protein monoclonal antibody 3H5 and counted using a fluorescent microscope, and percent of cells infected calculated. Data are expressed as mean and SD of triplicate wells. Representative data from one of multiple donors are shown. (C) The titre of infectious virus in the cell supernatant at 48 h post infection was determined by plaque assay. Due to variation between donors, the titre at 48 h in the absence of block was normalised to 100. Each data point is the average of three infected wells, each plaqued in triplicate, and the results from six donors are shown in this graph (mean represented by a bar). The p value was calculated by unpaired, two-tailed *t* test.

The functional role of MR in DV infection of primary human MØ was investigated using a polyclonal anti-MR antibody to block infection. This was examined in the IL-4-treated MDMØ since these cells showed the highest rate of DV infection. Anti-MR antibody significantly blocked DV infection of IL-4-treated MDMØ (p = 0.008) in all donors tested (6 donors; Figure 7A shows data from one representative donor). Normal goat serum control did not inhibit infection, suggesting that the MR may be a new functional receptor contributing to DV infection of human MØ. Production of infectious virus (pfu/ml) by these cells at 2 d post infection was reduced by 60%–95% with either mannan or anti-human MR antibody (Figure 7C), indicating that attachment and/or entry via this receptor is required for productive infection. The goat anti-human MR antibody blocked mannosylated BSA-FITC binding to both IL-4-treated MDMØ and 3T3 cells transfected with human MR (Figure S1).

Mannan, which blocks MR, DC-SIGN and other receptors with specificity for mannose, also blocked DV infection of MDMØ (Figure 7A and 7B). We tested the ability of several different anti-DC-SIGN antibodies to block infection of MDMØ. Antibodies against DC-SIGN have been shown by others to block DV infection of DC [3,4]. Interestingly, some anti-DC-SIGN monoclonal antibodies also blocked DV infection of IL-4-treated MDMØ (Figure 7B), and to a similar degree to that observed blocking with mannan or anti-MR antibodies.

## Discussion

We have shown for the first time that MR is a functional receptor for DV infection of human MØ. Binding of the MR to DV surface glycoproteins was mediated via the lectin activity of the CRD binding to glycans on the DV E protein. Gain of function binding data showed that surface expression of human MR on 3T3 cells was sufficient to confer DV binding. Antibodies specific for the MR significantly blocked both infection of MDMØ and the production of infectious virus in these cells. FACS analysis showed surface MR expression increased over 4-fold following IL-4 treatment of monocytes, corresponding with a similar fold increase in percent infected cells. Thus, the MR provides a potential link explaining the increase in MØ permissiveness to DV when stimulated by IL-4 or IL-13. We hypothesise that the MR may play a role in at least one of the stages of DV infection of human MØ. The first stage of virus entry into a cell is attachment, and the mechanism by which MR enhances the efficiency of DV entry could be by increasing virus attachment, as suggested for DC-SIGN and DV. As the MR can be internalized by macropinocytosis, pinocytosis, receptor-mediated endocytosis and phagocytosis, its role could also be in increasing the rate of DV internalisation, the second stage of virus entry. Analogous to the proposed mechanism of Fc-receptor enhancement of DV-antibody complex attachment/uptake in antibody-dependent enhancement, the presence of receptors such as MR or DC-SIGN, which enhance virus attachment/entry, may play a significant role in vivo.

Anti-DC-SIGN monoclonal antibodies were also able to block DV infection of MDMØ, as has been seen previously in MDDC. The anti-DC-SIGN antibodies that blocked DV infection of IL-4 treated MDMØ to the greatest degree (DC28 and 120612) are known to cross react with DC-SIGNR. We hypothesise that the different specificities of the monoclonal antibodies may explain why some but not all block DV infection of MØ. These findings corroborate data by Tassaneetrithep et al. [4] examining blocking of DV infection of THP-1 cells transfected with either DC-SIGN or DC-SIGNR, where the same two anti-DC-SIGN antibodies (but not others) blocked infection. The observation that antibodies to either MR or DC-SIGN can inhibit infection to such an extensive degree suggests that DV is likely to be using both MR and DC-SIGN for entry on cells that express both. It is difficult to assess the relative (and possibly differing) roles of MR and DC-SIGN in DV infection of primary MØ or DC that express both; however, our observation that expression of MR on 3T3 cells confers DV binding suggests that MR can mediate direct recognition of DV by myeloid cells. DC-SIGN has been suggested to function in DV attachment rather than internalisation, raising the possibility of another receptor being involved in the internalisation step of infection. At this stage the simplest hypothesis that explains the above findings in primary cells expressing both MR and DC-SIGN is that DC-SIGN is required for DV attachment and MR for internalisation. Alternatively, these receptors may function simultaneously and even co-operatively throughout the infectious process. Further studies on the cellular expression, location in the cell and ligand specificity of these receptors may provide clues as to the absolute roles they play in DV infection.

MR is ideally poised to act as a DV entry receptor given its constitutive recycling to the cell surface and ability to promote ligand internalisation via both endocytic and phagocytic pathways. While DC-SIGN mainly localises to the plasma membrane [22], ∼85% of cellular MR is located within the endocytic pathway [8] as a large intracellular receptor pool from which internalised receptor is rapidly replaced. In addition to expression on MØ, certain subpopulations of DC, including dermal DC in human skin, express the MR [22], in which case it may be involved in antigen delivery for presentation [23,24]. MR is expressed on MØ as well as cells lining venous sinuses in human spleen [25] and is therefore well located to act as a receptor for DV replication in these physiologically relevant target cells. Here it may have roles in clearance or adhesion, but also potentially in vascular leakage. A soluble form of MR has been found to be abundant in mouse [26] and human plasma (unpublished observations, L. Martinez-Pomares) and its CR domain has targets in the spleen. Binding of ligands by soluble MR can result in the transport of MR ligands to the B cell follicles, which may lead to clearance or enhanced presentation of viral antigen depending on TLR co-stimulation. Soluble MR could also potentially play a role in protection of virus from complement activation. Further elucidation of the exact role of MR in the attachment/entry/infectivity of DV will be a fundamental step in gaining a better understanding of DV pathogenesis.

MR and DC-SIGN both contain lectin domains, but differ distinctly in terms of ligand specificity, with MR binding terminal mannose, fucose and N-acetyl glucosamine, and DC-SIGN binding mannose within high-mannose oligosaccharides and fucosylated glycans [8,27,28]. Glycosylation endows unique properties to glycoproteins and can play a significant role in immunity. Recent observations using DV mutants in one or both of the N-linked glycosylation motifs have shown that N-linked glycosylation at Asn-67 is required for virus growth in mammalian cells [29]. In addition, new data by Mondotte and colleagues showed that DV lacking carbohydrate at Asn-67 had reduced capacity to infect MDDC [30]. This, combined with the observation that MDDC express both DC-SIGN and MR [22], and our demonstration that the presence of MR alone is sufficient to confer DV binding to transfected cells, suggest that glycosylation at Asn-67 may be relevant for mediating MR binding, in addition to that of DC-SIGN. While the sE may contain sulphated glycans, it was not bound by the CR fusion protein (Figure 1B), and so the CR domain of the MR is not expected to contribute to the binding of MR to DV. Our deglycosylation studies on sE show that it bears either complex or hybrid N-linked glycans. Terminal fucose is a reported ligand of MR and as such is the likely ligand on this source of DV antigen. A more detailed glycan analysis of DV grown in human MØ will be an important challenge for the future.

We expanded our study of the interaction of MR with DV by demonstrating binding of CRD4-7-Fc to all four serotypes of DV. Differences in glycosylation between DV serotypes, and more broadly between different flaviviruses, may be relevant for interaction with lectin receptors such as MR and DC-SIGN. We showed that MR can bind in ELISA to Japanese encephalitis virus and tick-borne encephalitis virus, both of which are reported to have glycosylated envelope proteins.

In this report we have shown that the type 2 cytokines IL-4 and IL-13 enhance the susceptibility of MDMØ to DV infection. The mechanisms resulting in increased infection in response to IL-4 and IL-13 are unknown. Analysis of IL-4-treated human monocytes showed that these cells are characterised by the overexpression and enhanced function of several endocytic receptors, including scavenger and C-type lectin receptors [18]. Functional ligand binding and transcriptional profiling studies [19,31] reveal that the MR is markedly upregulated on alternatively activated MØ.

A number of important conditions result in polarised activation of MØ phenotype. Our findings make it highly relevant to understand the clinical and epidemiological significance of a Th2 environment on DV infection, pathogenesis, and enhancement and in the development of a desirable vaccine response. It will be of great interest to examine the broader context in which dengue pathogenesis occurs by considering the effects on DV disease of settings that induce Th2 cytokines, including co-infection with parasites, the presence of immune complexes and allergy (e.g. asthma). There are few studies into the implications of co-incidence of parasitic infections or allergic disease and dengue infection. Guzman and colleagues showed significantly enhanced replication of DV in PBMC from asthmatic patients compared with controls [32]. During secondary dengue infection when anti-DV antibody is present, the study of DV interactions with MØ stimulated through FcγR ligation (‘type II' activated MØ), may be of particular relevance. Given the risk of antibody-dependent enhancement to vaccine trials, a Th2 environment (which is known to influence humoral responses and FcR expression [33,34]) may be a contributing factor to vaccine efficacy and DV pathogenesis and as such will require further examination.

## Materials and Methods

### Viruses and cell lines.

16681 and New Guinea C (NGC) strains of DV2 (both gifts from E. Gould, Oxford Centre for Ecology and Hydrology, UK) were propagated in the Aedes albopictus–derived C6/36 cell line (a gift from Armed Forces Research Institute of Medical Sciences, Thailand). Virus titres were obtained by plaque assay on LLC-MK2 monkey kidney cells (a gift from Armed Forces Research Institute of Medical Sciences, Thailand), as described previously [35]. 16681 strain DV2 grown in Vero cells, precipitated with polyethylene glycol, purified on a sucrose gradient and inactivated with formaldehyde was from Biodesign. Hawaii strain DV1, NGC strain DV2, H-87 strain DV3 and H-241 strain DV4 grown in C6/36 cells were precipitated with 7% polyethylene glycol and inactivated with beta-propiolactone (Biodesign). Human 293T-HEK cells (ATCC) were maintained in Dulbecco's modified Eagle's medium (DMEM) (Invitrogen) supplemented with 2mM glutamine, 0.1mg/ml streptomycin, 100U/ml penicillin and 10% heat inactivated fetal calf serum (FCS).

A stable NIH3T3 cell line expressing the human MR (3T3.hMR) was a gift from Gordon Brown (University of Cape Town, South Africa) and Philip Taylor (University of Cardiff, UK), made using the protocol described previously [21]. Human MR was amplified from cDNA derived from human mRNA. High expressing clones were selected after limiting dilution in 96 well plates. Control 3T3 cells expressing the vector only were prepared in parallel. Production of stable NIH3T3 expressing the murine MR (3T3.mMR) has been described previously [21]. These NIH3T3 transfectants were maintained in DMEM (Invitrogen) supplemented as above and with 0.6mg/ml geneticin (Invitrogen). NIH3T3 cells transfected with DC-SIGN (3T3.DCSIGN) were obtained through the NIH AIDS Research and Reference Reagent Program (from Drs Thomas Martin and Vineet KewalRamani) and maintained in similar DMEM medium without geneticin.

### Reagents.

Fc chimeric proteins (derived from the murine MR) and the hemagglutinin-tagged form of MR (MR-HA) were prepared by Richard Stillion as described previously [7,36]. Mannan was from Saccharomyces cerevisiae (Sigma). Antibodies were specific for DV2 E protein (3H5; a gift from Dale Greiner), MR (goat anti-hMR; a kind gift from Philip Stahl (Washington University School of Medicine, St. Louis, MO), DC-SIGN (120507), DC-SIGNR (120604) and both DC-SIGN and DC-SIGNR (DC28 and 120612) (all from R&D Systems). The specificity of anti-hMR antibody is examined in Figure S1.

### Preparation of primary cells.

For the generation of MDMØ and MDDC, human PBMC were isolated from buffy coats (NHS Blood and Transport) by centrifugation over a Ficoll-PaqueTM PLUS (Amersham) gradient, according to standard protocols. Adherent monocytes, isolated as described previously [37], were cultured in X-VIVO medium (BioWhittaker) with 1% heat-inactivated autologous plasma to allow differentiation into MDMØ. These cells were >95% macrophages phenotypically (CD14+, CD16-, CD86+, HLA-DR+, CD3−) (data not shown). For the generation of MDDC, recombinant human IL-4 (25ng/ml; Peprotech) and GM-CSF (50ng/ml; Peprotech) were added to monocytes in X-VIVO medium (BioWhittaker) with 1% heat-inactivated autologous plasma and the cells cultured for 4 d. The use of human blood was approved by the central Oxford University research ethics committee (MSD/IDEC/C1/2006/32).

### DV infection.

Monocytes were treated with recombinant human IL-4 (25ng/ml; Peprotech) or IL-13 (10ng/ml; Peprotech) for 2 d then infected with mosquito cell-grown 16681 DV2 virus. After 1 h the viral supernatant was replaced with cell culture medium without cytokine and the cells incubated for 2 d then fixed with 4% paraformaldehyde. In some experiments the IL-4-treated MDMØ were incubated with various blocking reagents for 40 min at 37°C before the addition of 16681 DV2 virus (in the presence of blocking agent).

### Detection of MR fusion protein binding to DV antigen ELISA.

96-well Maxi-Sorp plates were coated overnight with DV antigens (50ug/ml C6/36-grown NGC DV2 and 20ug/ml sE, or as specified in Figure 1) in phosphate-buffered saline (PBS; 138mM NaCl, 2.7mM KCl, 8mM Na_2_HPO_4_, 1.5mM KH_2_PO_4_, [pH 7.4]) at 4°C in triplicate. Wells were blocked with 0.5% immunoglobulin-free BSA (Sigma) before incubation with 15 ug/ml MR-HA, 2ug/ml human Fc-fusion MR domain proteins or 15ug/ml rabbit anti-DV1–4 antibody for 2 h at room temperature in Tris buffered saline (TBS; 10mM TrisHCl, pH 7.4, 0.154 M NaCl, 0.05% Tween 20) containing 10mM CaCl_2_. For competitive binding studies MR fusion proteins were pre-incubated for 30 min on ice in TBS containing 1M NaCl and 10mM CaCl_2_ in the presence of 2mM d-mannose, 2mM l-fucose or 2mM galactose, or TBS containing 10mM EDTA. The wells were washed 6 times and binding of the Fc-fusion proteins was detected by incubating the wells with an alkaline phosphatase-conjugated anti-human antibody (1:1,000 dilution), visualized using 1mg/ml 4-Nitrophenyl phosphate disodium salt hexahydrate in 100mM Tris, 100mM NaCl, 5mM MgCl_2_, pH9.5, and the absorbance was read at 405nm. The wells with HA-tagged protein were incubated with 10ug/ml of MR5D3 (rat anti-mouse MR; [21]) for 1 h at room temperature followed by an alkaline phosphatase-conjugated anti-rat antibody (1:1,000 dilution) and visualized as above. The wells with rabbit anti-DV1–4 were incubated with an alkaline phosphatase-conjugated anti-rabbit antibody (1:1,000 dilution) and visualized as above. Wells coated with mannan and SO_4_3GalNAc-PAA (Lectinity) were included as a positive control for proteins containing the CRD4–7 and CR domains respectively (data not shown). We verified that DV2 ligands had coated all of the wells equally by using direct ELISA detection of DV antigens 3H5 (10ug/ml) followed by a goat anti-mouse alkaline phosphatase-labelled (1:1,000 dilution) secondary antibody and visualised as above.

### Blot overlays.

CRD4–7 binding to sE was investigated by blot overlay by running 0.9ug sE protein and 0.9ug sE protein deglycosylated with peptide: N-glycosidase F (New England Biolabs) on 10% SDS-PAGE, and subsequently transferring proteins to Hybond C-Extra nitrocellulose membranes (Pharmacia). Blots were blocked for 1 h in 0.5% skimmed milk powder in TBS containing 10mM CaCl_2_ (blocking/washing solution). Blots were probed with 1ug/ml MR CRD4–7-Fc in the absence or presence of either 2mM d-mannose, 2mM l-fucose or 20mM EDTA for 2 h, and then washed three times with blocking solution. Binding was detected with 1ug/ml horseradish peroxidase-conjugated anti-human IgG antibody (Vector Laboratories) and visualized by chemiluminescence. Blots probed in the presence of inhibitors were washed 3 times and reprobed with MR CRD4–7-Fc in the absence of inhibitor. Blots were stripped by incubation in 63mM Tris, pH 6.7, 2% SDS and 100mM 2-mercaptoethanol at 50°C for 30 min, re-blocked and probed with the anti-DV E-protein antibody, 3H5, at 10ug/ml. Binding was detected with 10ug/ml horseradish peroxidase-conjugated anti-mouse IgG antibody and visualized by chemiluminescence.

### Production of recombinant sE protein.

An open reading frame consisting of the last 20 aa of C-protein, the entire prM protein, and E protein truncated by 96 amino acids at the C-terminus and containing a hexahistidine tag was amplified by PCR from cDNA prepared from DV2 strain 16681. The expression cassette was cloned in the mammalian expression vector pLEX [38] and transfected into human 293T-HEK cells cultured in Optimem (Invitrogen), and sE was partially purified from the supernatants of these cultures by Ni-chelation affinity chromatography (Figure 3A). The identity of the protein was confirmed by mass-spectrometric analysis of peptides resulting from tryptic digest of the excised SDS-PAGE band (data not shown).

### sE glycan analysis.

Glycans were released from approximately 25ug of recombinant soluble dengue virus E-glycoprotein and labelled by reductive amination with the fluorophore 2-aminobenzamide [39,40]. The glycans were processed through NP-HPLC and the retention times for the individual glycans were converted to glucose units (GU) using a standard dextran curve [41]. These were then compared with a database of experimental values (http://glycobase.ucd.ie/cgi-bin/public/glycobase.cgi) and initial assignments made were confirmed following digestions of the glycans with an array of exoglycosidase enzymes [40].

### Flow cytometry.

FACS was performed according to conventional protcols at 4°C in the presence of 2mM NaN_3_. Non-specific binding sites on cells were blocked with PBS containing 5% heat-inactivated rabbit serum, 5% heat-inactivated goat serum, 0.5% BSA and 5mM EDTA (blocking buffer) before the addition of primary antibodies. Surface expressed MR and DC-SIGN was detected using 10ug/ml 15–2 (Serotec) and 120507 (R&D Systems) monoclonal antibodies, respectively, and was compared with an isotype control (Serotec). Surface bound DV was detected using 10ug/ml 3H5. The primary antibodies were detected using Alexafluor 488-conjugated anti-mouse antibody (Molecular Probes) diluted 1:200 in blocking buffer. Cells were fixed with 1% paraformaldehyde in PBS before analysis. Binding was quantified on a FACSCalibur flow cytometer and data from ∼10,000 cells were routinely acquired for each sample. Data were analysed using FlowJo software (Treestar). Percent of max represents the number of events normalised according to FlowJo algorithms. Fold increase in receptor expression was measured by geometric mean fluorescent intensity of specific receptor antibody staining for IL-4-treated cells divided by untreated cells.

### Cell binding assays.

Cells suspensions were prepared by scraping cells to preserve receptor expression at the cell surface. Mosquito cell-grown NGC DV2 (1.5 × 10^6^ pfu/ml) or media was incubated with cells (4 × 10^6^) at a multiplicity of 0.35 infectious virions per cell for 80 min on ice. Unbound virus was washed away with cold media, the cells were fixed with 1% paraformaldehyde in PBS, and surface bound virus detected with anti-DV E-protein antibody by flow cytometry as described above.

FITC-labelled, mannosylated or galactosylated BSA (5ug/ml; Sigma) was incubated for 90 min at 37°C with primary human MDMØ or 3T3 transfectants plated on tissue culture-treated plastic. For blocking studies cells were pre-incubated with mannan (2mg/ml), normal goat serum (NGS) or goat-anti hMR antibody for 20 min at 37°C. After incubation cells were washed with PBS, harvested using PBS containing 5mM EDTA and lidocaine (4mg/ml) and fixed in 2% paraformaldehyde in PBS. Binding was quantified by a FACSCalibur flow cytometer and analysed using FlowJo software.

### Immunofluorescence microscopy.

Fixed, DV-infected cells were permeabilised with 0.5% Triton-X and stained with 10ug/ml 3H5 followed by an Alexafluor 488-labelled secondary anti-mouse IgG antibody (Molecular Probes) and the nuclei stained with DAPI. Stained coverslips were mounted in DakoCytomation fluorescent mounting medium (Dako), and analyzed using either CCD1 (Axioplan) and CCD camera (Spot) or a META^TM^ confocal microscope linked to LSM 510^TM^ software (Carl Zeiss MicroImaging, Inc.). Confocal images were acquired sequentially using the multitrack configuration of the Zeiss META^TM^ to avoid bleed-through between fluorescence channels, and the appropriate controls with and without primary antibody were performed. Additional image processing was performed using Adobe Photoshop 7. The image is presented as single two-dimensional *x-y* sections and the corresponding transmission image. At least twelve fields (600–1,000 individual cells) were counted by fluorescent micrsocopy.

### Statistical analysis.

Statstics were calculated using GraphPad PRISM (version 2.0; GraphPad Software, San Diego, CA) and Microsoft Excel. Two-tailed Student's *t* tests were used to calculate p values.

## Supporting Information

Figure S1 Goat Anti-Human MR Antibody Specifically Blocks Mannosylated BSA-FITC Binding to Cells Expressing Human MR (2.1 MB TIF)

Table S1 Effects of Cytokines and Cytokine Combinations on DV Infection of Monocytes (35 KB RTF)
