# Correction: The Mannose Receptor Mediates Dengue Virus Infection of Macrophages

**DOI:** 10.1371/annotation/98b92fca-fa6e-4bf3-9b39-13b66b640476

**Published:** 2008-03-21

**Authors:** Joanna L Miller, Barend J. M de Wet, Luisa Martinez-Pomares, Catherine M Radcliffe, Raymond A Dwek, Pauline M Rudd, Siamon Gordon

The surname of co-author Barend J. M. de Wet was misspelled in the originally published article.

