# Supplementary material for: The Mannose Receptor Mediates Dengue Virus Infection of Macrophages
Source: PLoS Pathog. 2008 Feb 8;4(2):e17. doi: 10.1371/journal.ppat.0040017 (PMC2233670; doi:10.1371/journal.ppat.0040017)
Supplement: Supplementary file 1 [file ppat.0040017.st001.rtf]

Supp. Table 1: Effects of cytokine and cytokine combinations on DV infection of monocytes^a^. 
Cytokine/treatment
(concentration in monocyte culture medium)	Percent cells infected with DV	
Serum free	4.7	
1% human plasma	4.9	
GM-CSF (50 ng/ml)	2.5	
M-CSF (50 ng/ml)	1.8	
IL-4 (25 ng/ml)	13.8	
IL-3 (25 ng/ml)	1.7	
M- and GM-CSF	1.7	
IL-13 (10 ng/ml)	7.4	
IL-6 (10 ng/ml)	4.5	
IL-10 (10 ng/ml)	2.6	
TGFβ (10 ng/ml)	2.2	
IFNγ (20 ng/ml)	1.8	
IFNα (100 U/ml)	2.7	
LPS (50 ng/ml)	2.6	
IL-5 (10 ng/ml)	2.6	
TNFα (10 ng/ml)	4.4	
PGE_2_ (100 ng/ml)	3.9	

^a^ Human monocytes were incubated with each cytokine or combination of cytokines for 48 hr in medium containing 1% heat-inactivated autologous plasma (except for 'serum free' condition) prior to infection with DV2 at a multiplicity of infection of 0.5. 2 days following infection the cells were fixed, permeabilised and stained with antibody to dengue E protein followed by an Alexa488-labelled secondary antibody and DAPI. The percentage of infected cells was calculated from counts of infected cells and total cells. Table shows data from a single donor however treatment with IL-4/IL-13 was repeated with multiple donors (at least 6 donors).
